# Longevity and How to Obtain It

**Published:** 1907-06

**Authors:** 


### LONGEVITY AND HOW TO OBTAIN IT.

This is the title of a very interesting paper in the London Lancet by Sir L. Brunton. He considers the nature of the risks which old people run and how they may be best foreseen and averted so that great activity in advanced years may become the rule instead of the exception. These risks may be divided into: (a) those which arise from external influences, and (b) those which originate in the body itself. Of course, the action of both classes of causes may be combined. The introduction of antiseptic methods in surgery is reducing the number of deaths from septic infection and so increasing longevity. This is especially true as regards the disease of the bladder and the introduction of prostatectomy. Erysipelas is a common cause of death among the aged, and should be diminished greatly by antiseptic measures. Respiratory diseases carry off many aged persons; while they are usually consequent upon exposure to cold, yet pathological organisms play the most important part. Many so-called common colds are very infectious and readily transmitted. Dust is a most important factor in the causation of colds. So that aged people should be protected from dust, from cold and from sudden changes of temperature and draughts. Wind fairly in the face is little to be dreaded, but at the back of the neck or at the side of the head it is much more dangerous. But one of the most important methods of securing health is to increase the patient's power of resistance. The peripheral vessels of the whole body should be trained to alternate heat and cold; this is best done by daily baths, exercise and walking. In old age intestinal diseases of bacterial origin are comparatively rare. Cancer is a common cause of death among the aged. We cannot say at present how much faulty nutrition has to do with this. But diet has a most important effect upon diseases of the circulation, which are more fatal among the aged than ever cancer. Arteriosclerosis, with its attendant evils, is the great enemy to longevity. And arteriosclerosis is the result of heightened blood pressure, which in turn is most often due to faulty diet, excessive use of meat, strong soups, etc. Keep down the blood pressure and you prolong life. Cholagogues and purgatives, potassium iodide, the nitrites and nitrates are all useful to this end. In our opinion such medication is useless and even dangerous unless specially indicated. More can be done for the aging by drinking buttermilk than by any medicinal means.—Medical Council, May, 1907.

